# 1831. Longitudinal Changes in Antimicrobial-resistant Bacterial Bloodstream Infections in the US Military Health System from 2010-2019

**DOI:** 10.1093/ofid/ofac492.1461

**Published:** 2022-12-15

**Authors:** Alexander C Vostal, Melissa Grance, John H Powers, Leigh Carson, Uzo Chukwuma, Charlotte Lanteri, Nicholas Seliga, Beth Poitras, Edward Parmelee, Katrin Mende

**Affiliations:** National Institute of Allergy and Infectious Diseases, Silver Spring, Maryland; Infectious Disease Clinical Research Program, Department of Preventive Medicine and Biostatistics, Uniformed Services University of the Health Sciences, Bethesda, Maryland; Leidos Biomedical Research, Rockville, Maryland; Infectious Disease Clinical Research Program, Bethesda, Maryland; Navy and Marine Corps Public Health Center, Rockville, Maryland; Infectious Disease Clinical Research Program, Department of Preventive Medicine and Biostatistics, Uniformed Services University of the Health Sciences, Bethesda, Maryland; Navy and Marine Corps Public Health Center, Rockville, Maryland; Navy and Marine Corps Public Health Center, Rockville, Maryland; Infectious Disease Clinical Research Program, Department of Preventive Medicine and Biostatistics, Uniformed Services University of the Health Sciences, Bethesda, Maryland; Infectious Disease Clinical Research Program, Department of Preventive Medicine and Biostatistics, Uniformed Services University of the Health Sciences, Bethesda, MD, USA, San Antonio, Texas

## Abstract

**Background:**

The epidemiology of antibiotic-resistant pathogens guides antimicrobial therapy for bacterial bloodstream infections (BSI). We describe changes in antimicrobial-resistant BSI pathogens over time within the US Military Health System (MHS), which prospectively captures clinical and microbiological data from both retired and active-duty US Uniformed service members and their beneficiaries.

**Figure:**
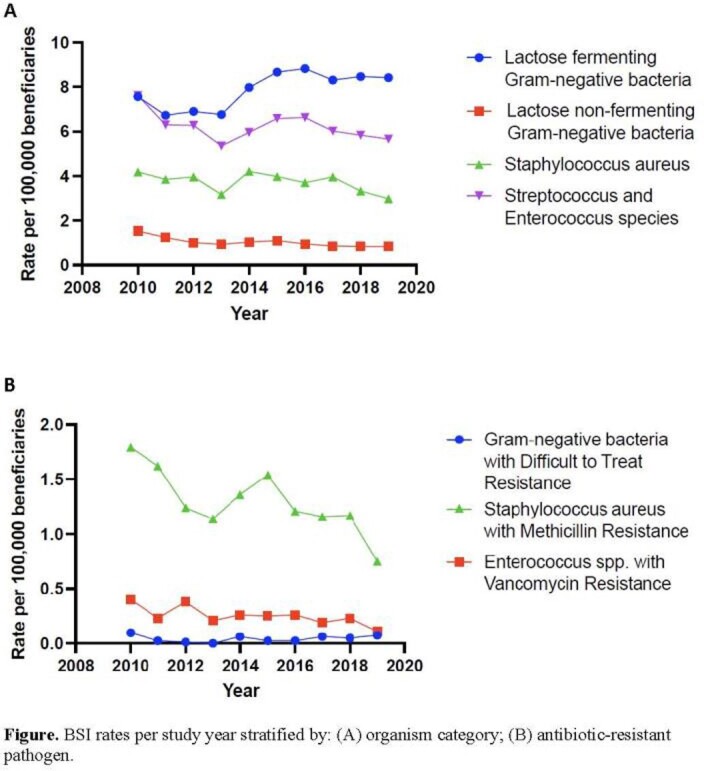

**Methods:**

The study population included MHS beneficiaries with blood cultures positive for any bacterial pathogens (Jan 2010 – Dec 2019). Microbiological data were obtained from the Navy and Marine Corps Public Health Center and antibiotic resistance was interpreted using CLSI breakpoints corresponding to collection year. Blood contaminants were excluded. Difficult to treat resistance (DTR) was defined in Gram-negative bacteria (GNB) as isolates with *in vitro* resistance to three classes of antibiotics: carbapenems, extended-spectrum cephalosporins, and fluoroquinolones.

**Results:**

The 15 most frequent bacterial pathogens, representing 15,358 BSI episodes from 12,749 individuals, were subcategorized in four groups based on shared BSI clinical features. Lactose-fermenting GNB (LFGNB) were most common, accounting for 42% of BSI pathogens, following by *Streptococcus/Enterococcus* spp. (33%), *Staphylococcus aureus* (20%), and non-lactose fermenting GNB (NLGNB, 5.5%). The rate of LFGNB BSI increased from 7.57 per 100,000 beneficiaries in 2010 to 8.42 in 2019 (peak of 8.83 in 2016), resulting in an increase of 11.3% during the study period (Figure). Rates of BSI attributed to *Streptococcus/Enterococcus* spp., *S. aureus*, and NLGNB decreased 26%, 29%, and 45%, respectively, over the study period. The average annual rates of methicillin-resistant *S. aureus*, vancomycin-resistant *Enterococcus* spp., and DTR GNB BSI were 1.30, 0.25, and 0.05 per 100,000 beneficiaries, respectively. Over the study period, these rates decreased 58.3%, 72.4% and 24.2%, respectively.

**Conclusion:**

LFGNB BSI numerically increased over time while NLGNB BSI (e.g., *Pseudomonas aeruginosa* and *Acinetobacter* spp.) decreased. The burden of DTR GNB BSI also decreased, indicating that first-line antibiotics remain clinically available for most patients with BSI.

**Disclosures:**

**John H. Powers, III, MD**, Arrevus: Advisor/Consultant|Eicos: Advisor/Consultant|Evofem: Advisor/Consultant|Eyecheck: Advisor/Consultant|Gilead: Advisor/Consultant|GlaxoSmithKline: Advisor/Consultant|OPKO: Advisor/Consultant|Resolve: Advisor/Consultant|Romark: Advisor/Consultant|SpineBioPharma: Advisor/Consultant|UTIlity: Advisor/Consultant|Vir: Advisor/Consultant.

